# A high throughput reporter virus particle microneutralization assay for quantitation of Zika virus neutralizing antibodies in multiple species

**DOI:** 10.1371/journal.pone.0250516

**Published:** 2021-04-23

**Authors:** Kelly Bohning, Stephanie Sonnberg, Hui-Ling Chen, Melissa Zahralban-Steele, Timothy Powell, Greg Hather, Hetal K. Patel, Hansi J. Dean

**Affiliations:** Takeda Vaccines, Inc., Cambridge, Massachusetts, United States of America; CEA, FRANCE

## Abstract

Zika virus is a Flavivirus, transmitted via *Aedes* mosquitos, that causes a range of symptoms including Zika congenital syndrome. Zika has posed a challenging situation for health, public and economic sectors of affected countries. To quantitate Zika virus neutralizing antibody titers in serum samples, we developed a high throughput plate based Zika virus reporter virus particle (RVP) assay that uses an infective, non-replicating particle encoding Zika virus surface proteins and capsid (CprME) and a reporter gene (*Renilla* luciferase). This is the first characterization of a Zika virus RVP assay in 384-well format using a Dengue replicon *Renilla* reporter construct. Serially diluted test sera were incubated with RVPs, followed by incubation with Vero cells. RVPs that have not been neutralized by antibodies in the test sera entered the cells and expressed *Renilla* luciferase. Quantitative measurements of neutralizing activity were determined using a plate-based assay and commercially available substrate. The principle of limiting the infection to a single round increases the precision of the assay measurements. RVP log_10_EC_50_ titers correlated closely with titers determined using a plaque reduction neutralization test (PRNT) (R^2^>95%). The plate-based Zika virus RVP assay also demonstrated high levels of precision, reproducibility and throughput. The assay employs identical reagents for human, rhesus macaque and mouse serum matrices. Spiking studies indicated that the assay performs equally well in different species, producing comparable titers irrespective of the serum species. The assay is conducted in 384-well plates and can be automated to simultaneously achieve high throughput and high reproducibility.

## Introduction

Zika virus (ZIKV), first identified in 1947, is a member of the *Flaviviridae* [1–3]. ZIKV is closely related to Dengue viruses and is transmitted by *Aedes aegypti* and *Aedes albopictus* mosquitoes, sexually, and vertically from mother to fetus [2, 3]. ZIKV circulated in Africa, Southeast Asia, and the Pacific regions before it was first detected in South America in 2015 [3]. Major outbreaks in the Americas in 2015, notably in Brazil, linked ZIKV infections with cases of congenital malformations in newborns, Guillain-Barré Syndrome (GBS) and other neurological complications in adults [3, 4]. As there is no specific preventative or treatment for ZIKV infection, there is an urgent medical need for ZIKV vaccines capable of preventing infections, particularly during pregnancy.

Vaccines against other flaviviruses such as Japanese encephalitis virus (JEV), yellow fever virus (YFV), and tickborne encephalitis virus (TBE) elicit neutralizing antibodies directed against the envelope (E) protein in vaccinated subjects and confer protection against disease [5–11]. Published studies support ZIKV neutralizing antibodies as a likely primary immunological endpoint and mechanism of immune-mediated protection from experimental ZIKV challenge [12–16].

The plaque reduction neutralization test (PRNT) or microneutralization test (MNT) approaches have been the gold standards for measuring neutralizing antibody responses against most flaviviruses, including YFV, JEV, and Dengue virus (DENV) [10, 17]. Both the MNT and PRNT performed in 96 well plates are largely manual, labor-intensive assays, which make them challenging and expensive to use in settings such as large clinical trials in which hundreds or thousands of samples need to be tested under good clinical laboratory practice conditions [18, 19]. In addition, both the PRNT and MNT assays rely on the use of infectious virus as a reagent to detect neutralizing antibodies and can have high variability among laboratories depending on the cells due to differences in cell lines used for viral growth, the strain, specific infectivity and maturation state of the virus reagent, and other variations in assay conditions [18, 20, 21].

Reporter virus particles (RVPs) have been used as a substitute for the live virus reagent in neutralization assays to measure antibodies against several flaviviruses including DENV, YFV, JEV, and West Nile Virus (WNV) [22–28], and most recently with ZIKV [16, 28, 29]. RVPs are replication-deficient and deliver their reporter gene to permissive cells [26–28, 30–32].

Flavivirus RVPs containing the virus surface antigens (prM/M and E) have been shown to be antigenically indistinguishable from infectious virus using virus-specific antibodies [26–28, 30–32]. DENV neutralizing monoclonal antibody (mAb) 4G2 neutralized all four serotypes of DENV-RVPs while non-neutralizing mAb 15F3 did not neutralize any [24]. DENV-2 specific neutralizing mAb 3H5 was shown to only neutralize DENV-2 RVP as expected [24]. WNV neutralizing mAb 7H2 also neutralized WNV-RVP [26].

To address some of the limitations of the traditional PRNT and MNT assays, we have developed a ZIKV RVP assay in 384-well plates (Z-RVP-384 assay), that is high throughput, automatable, does not use live ZIKV but rather an RVP that is capable of one round of infection only, and is reproducible. The Z-RVP-384 assay is applicable to measuring serum antibodies in humans and multiple other species. Here we report the development and optimization of the Z-RVP-384 assay and its performance characteristics.

## Results

### Overview of the Z-RVP-384 assay

The Z-RVP-384 assay detects ZIKV neutralizing antibodies in serum using ZIKV reporter virus particles (Z-RVP) that contain the structural proteins CprME from ZIKV strain SPH2015 and a *Renilla* luciferase reporter gene in a 384-well plate format. SPH2015 was selected as a representative of circulating ZIKV strains, as RVPs created from five ZIKV strains representing African (MR-766, ArB7701) and Asian (H/PF/2013, PHL/2012 and THA/2014) have been demonstrated to neutralize ZIKV convalescent serum to equivalent titers [29].

The principle stages of the assay are serum heat inactivation and serial dilution, addition of Z-RVP and neutralization at 37°C for 1 hour, addition of Vero cells, incubation at 37°C, 5% CO2, for 72 hours, addition of *Renilla* substrate and detection of chemiluminescence, and calculation of neutralization titers.

### Assay optimization

Previously described RVP assays and neutralization assays were used for guidance to determine key assay parameters for assay optimization and control [26–28, 30, 31] with the goal of developing a well-controlled and robust neutralization assay optimized for low assay variability, maximum dynamic range, and potential for high throughput. Critical parameters that were optimized included neutralization duration, RVP concentration, cell concentration, assay incubation time and assay medium.

#### Optimization of neutralization duration, cell concentration, assay incubation duration and assay medium

**Fig 1:**
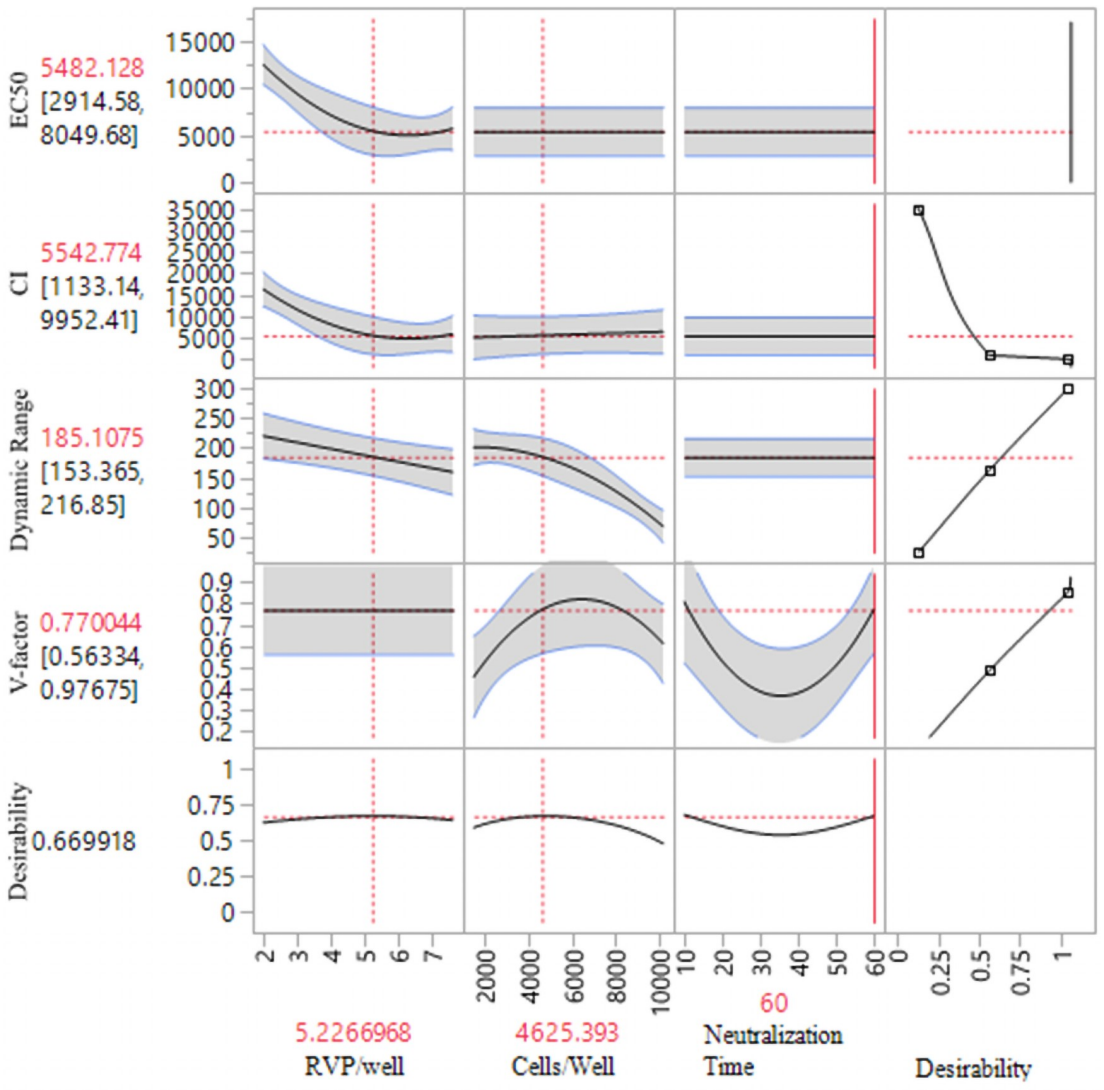
Predicted optimal combination of factors with maximal desirability. DOE analysis was carried out using the optimization and desirability settings (minimized with the relative importance set to 1. V-factor: maximized with the relative importance set to 0.7. Dynamic Range: maximized with the relative importance set to 1) in JMP13. The curves show the predicted interaction between the different factors. Flat lines indicates no predicted interaction. The horizontal redlines show the predicted optimal parameter and the vertical red lines show the geometric mean of the desirability measured.

A design of experiment (DOE; JMP^®^13. SAS Institute Inc., Cary, NC, 1989–2019) approach was used to facilitate optimization of Z-RVP-384 assay parameters (neutralization duration, cell concentration, assay incubation time, and assay medium). The goal of the optimization was to minimize assay variability, maximize dynamic range and maximize the signal in relative luciferase units (RLU). The effects of neutralization time (10 min, 35 min, 60 min), Z-RVP reagent per well (2 μL, 4.75 μL, and 7.5 μL), and number of Vero cells per well (1500, 5750, 10000) were investigated. Heat-inactivated ZIKV hyperimmune rabbit serum was diluted 1:5 in assay media then serially diluted 5-fold ten times. The experiment was then conducted according to 16 experimental combinations determined using a custom JMP 13 DOE method as described by JMP13 [33]. The DOE was then analyzed based on the EC50 titers and confidence limits, V-factor and dynamic range. The V-factor gives a realistic measure of the overall assay performance by accounting for intermediate points in the dose-response curve that have higher variability due to effects of computation and dispensing errors [34]. V- factor is calculated by using the formula $V-Factor=1-\;6\frac{mean\;(\sigma )}{\left|\sigma \rho -\sigma n\right|}$ where σ is standard deviation (SD) of the curve and σp is the SD for positive control and σn is SD for negative control [35]. A 4-parameter non-linear regression model was used to obtain the EC_50_ titers and confidence limits. The dynamic range was calculated using the formula: *Dynamic range* = *Average Max RLU signal* − *Average Min RLU signal*. The DOE stepwise modeling demonstrated that the Z-RVP amount per/well and the cells/well significantly impacted the assay. The next step was to set the “desirability” for each parameter to be optimized. The desirability is set by determining the goal of each response such as maximizing the dynamic range and V-Factor, minimizing the confidence limits and setting the “importance” to predict the neutralization time, Z-RVP amount and optimal number of Vero cells as shown in prediction profiler (Fig 1).

The prediction profiler determined the parameters which resulted in the lowest confidence limits and the highest V-factor and dynamic range as RVP/well: 5.2, cells/well: 4625 and neutralization time: 60 minutes. Optimized parameters were repeated in a confirmatory assay run. Next, we compared assay duration of 48 hours, and 72 hours. A 72 hour assay duration resulted in higher RLU of 397,000 as compared to a RLU of 331,000 after 48 hr. Optimized assay parameters were defined as neutralization for 60 min at 37°C, using 5.25 μL RVP of lot 164C (optimal RVP amount is reassessed for each lot), and 4625 Vero cells/384-well with an assay duration of 72 hours.

#### Z-RVP reagent assessment

The structure, antigenicity, stability and reproducibility of the Z-RVP reagent has been described [32]. The Z-RVP reagent was assessed for relative infectivity and cytotoxicity. The Z-RVP reagent was supplied as single-use aliquots and stored frozen at <-60°C. Immediately prior to use Z-RVP aliquots were rapidly thawed in a 37°C water-bath and kept on ice until use.

Akin to virus preparations, each lot of RVP has a different level of infectivity and therefore a standard infectivity assay was carried out to establish the input concentration of a specific lot of Z-RVP. Relative Z-RVP infectivity was assessed by titration from 7.5 μL to 0.5 μL per 384-well using serial dilution in assay medium alone. Neat or diluted Z-RVP (7.5 μL) was transferred to a 384-well plate and Vero cells in assay media added to a final volume of 22.5 μL/well. Plates were incubated in a humidified incubator at 37 ± 2°C and 5% (±1%) CO_2_ for 72 ± 2hrs and read using the *Renilla*-Glo Luciferase Assay System. Only Z-RVP lots that resulted RLU > 100,000 were used in subsequent experiments to ensure adequate dynamic range between full neutralization (no signal) and no neutralization (maximum signal).

**Fig 2:**
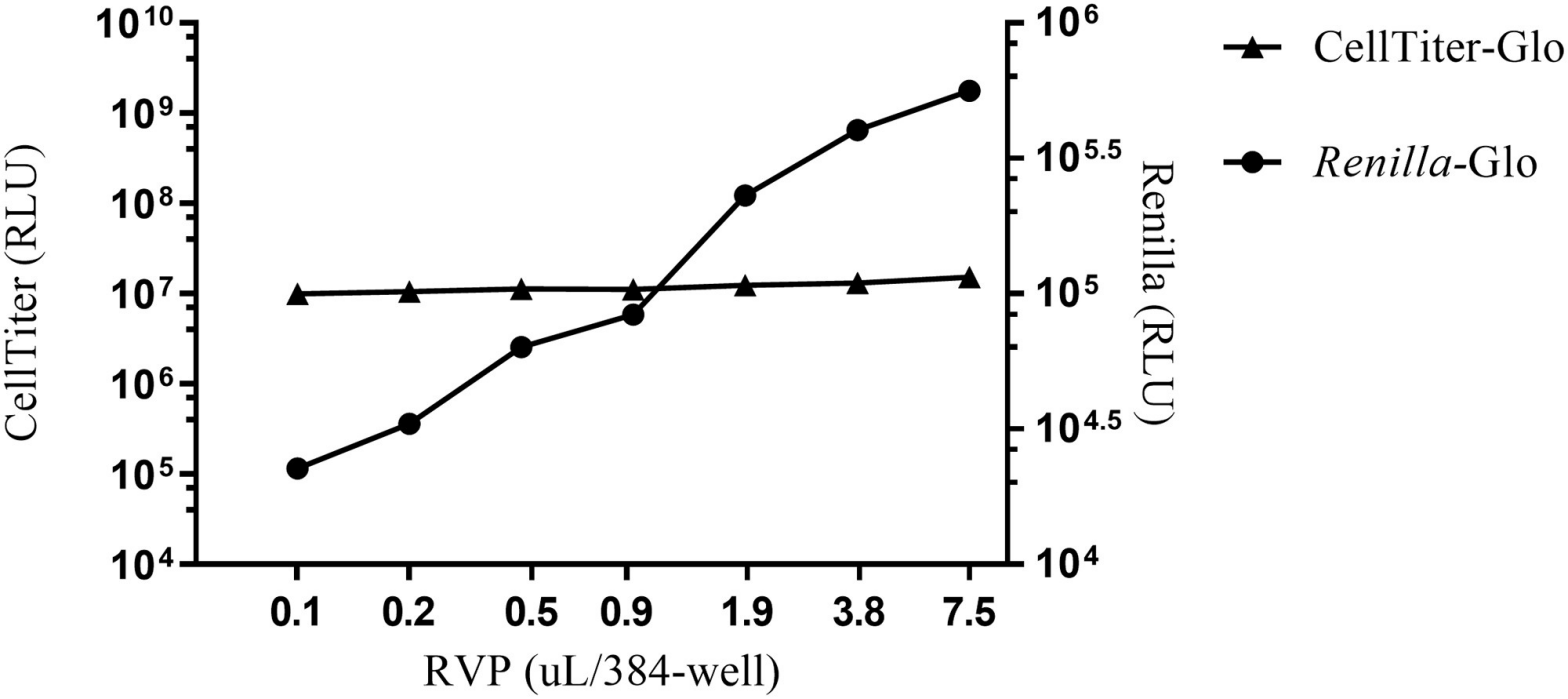
Viability and infectivity assessment for Z-RVP lot 229A. The CellTiter-Glo (RLU) response is displayed on the left y-axis and the *Renilla*-Glo (RLU) response is shown on the right y-axis. The CellTiter-Glo RLU expressed by the cells was consistent over Z-RVP amount tested whereas the *Renilla*-Glo (RLU) increases as the Z-RVP amount increases.

Potential cell toxicity of the Z-RVP reagent lot 229A on Vero cells plated at 4625 cells/well was assessed using the CellTiter-Glo detection System in parallel to the Z-RVP infectivity. The results of the CellTiter-Glo study demonstrate that Vero cells are viable at the optimal concentration of cells determined by the DOE (Fig 2). The RLU signal increased with increasing amount of Z-RVP. No toxicity of the Z-RVP reagent on cells was observed with the 16 lots of Z-RVP tested. Of these lots, 8 were selected for use in assay development.

#### Determination of optimal Z-RVP particle input

As the read-out of the Z-RVP-384 assay was chemiluminescence signal, the Z-RVP reagent was optimized towards both achieving a minimum required signal as well as observing the law of mass action: Antibodies in undiluted serum are to greatly outnumber Z-RVP particles to assure that binding of antibodies to the antigen do not change the dynamics of neutralization [31]. We established a minimum chemiluminescence signal of 100,000 RLU to allow for adequate range across the neutralization curves, with a maximum signal predetermined by the chemiluminescence reader capacity of 1x10^7^ RLU.

**Fig 3:**
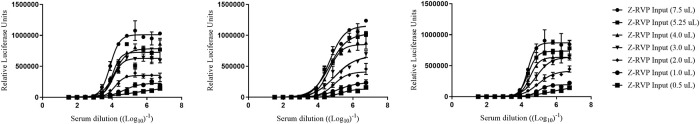
Neutralization curves of Z-RVP dilutions. Neutralization curves of different dilutions of Z-RVP to determine the effects of Z-RVP input against Serum 1, 2 and 3. Neutralization titers in log_10_EC_50_ titers are shown as calculated by nonlinear regression in GraphPad Prism for each Z-RVP dilution.

To determine the optimal Z-RVP input per well for the lot 229A Z-RVP lot was titrated from 0.5 μL to 7.5 μL in a neutralization assay using positive control antisera (three different rabbit sera, generated by immunizing with purified live ZIKV, strain PRVABC59 (Serum 1) or Dakar (Sera 2 and 3)). Neutralization curves were generated for the different Z-RVP dilutions as show in Fig 3. The log_10_EC_50_ titers for the three sera were then examined against input Z-RVP amount to determine a stable (similar) titer that was consistent with the titers produced using other Z-RVP lots. Z-RVP lot 229A input amounts of 2 μL and 3 μL resulted in similar log_10_EC50 values within the variation of the assay (0.2log_10_) for all three positive control sera (Table 1). The midpoint of 2.5 μL was chosen as optimal for this Z-RVP lot and assay conditions.

**Table 1:** Titration of Z-RVP reagent for use in the Z-RVP-384 assay.

RVP lot 229A input (μL)	Average RLU	Serum 1 Log_10_EC_50_	Serum 2 Log_10_EC_50_	Serum 3 Log_10_EC_50_
7.5	1,037,777	3.89	4.65	4.36
5.25	857,748	4.06	4.68	4.37
4	796,357	4.14	4.62	4.48
3	644,667	4.06	4.94	4.83
2	408,427	4.17	4.90	4.96
1	217,585	4.68	5.12	4.96
0.5	146,924	5.38	5.31	5.75

Determination of area of stable log_10_EC_50_ titers for three sera positive for ZIKV neutralizing antibodies using different input amounts of Z-RVP reagent (lot 229A). Z-RVP lot 229A input amounts of 2 μL and 3 μL resulted in similar log_10_EC_50_ values within the variation of the assay (0.2log10) for all three positive control sera.

#### Signal stability, replication of measurements, and plate layout

The stability of the *Renilla* RLU signal in 384-well plates was assessed through an interleaved assay to determine uniformity and separation of signals to determine edge effect, drift or other spatial effects present in the assay as outlined in the Assay Guidance Manual by Eli Lilly & Company and the National Center for Advancing Translational Sciences [35]. The same operator carried out duplicate tests on two separate days (day 1, day 2) of maximum (max), mid, and minimum (min) RLU generating serum samples in different positions. The maximum RLU serum sample was generated by diluting negative rabbit serum in assay media at a 1:1607.5 dilution. The mid RLU serum sample was generated by diluting ZIKV positive rabbit serum against ZIKV strain PRVABC59 in assay media at a 1:1607.5 dilution. The minimum RLU serum sample was generated by diluting the ZIKV positive rabbit serum against ZIKV strain PRVABC59 in assay media at a 1:5 dilution. For each max, medium and min RLU on the plate the mean RLU, standard deviation (SD) and Coefficient of Variation (CV) were determined. The % RLU midpoint as a surrogate EC50 between the three serum dilutions was calculated by the following equation: ($\%RLU\;midpoint=\frac{well(mid)-AVG(min)}{AVG(max)-AVG(min)\;}\times 100$). AVG (min) was the average taken over the three minimum signal plate averages, and AVG (max) was the average taken over the three maximum signal plate averages [35]. The % RLU midpoint assessed the stability of the RLU midpoint while allowing for variation between max and min RLUs between plates. Acceptance criteria were based on the recommendations specific to high throughput screening assay validation in the Assay Guidance Manual by Eli Lilly & Company and the National Center for Advancing Translational Sciences [35]. The following acceptance criteria were established: CV (of the mean) ≤ 20%, SD midpoint < 20%, RLU (signal) window ≥ 2, Z’ factor ≥ 0.4, drift or edge effects < 20%.

The % midpoint RLU activity was consistent between occasions with a less than 2-fold difference in midpoint between plates (between plates; between occasions). The acceptance criteria for inter-plate and inter-day tests were met. The mean CV of the RLU signal by sample, plate, and day was assessed. A significant edge effect of rows A and P was identified, and these rows were subsequently excluded from use and filled with assay media. Similarly, the edge columns 1 and 24 were excluded from use leaving the central plate area containing 308 wells (14 rows and 22 columns). A plate layout was established: 12 test samples, 2 controls (1 negative serum, 1 positive serum) each titrated in eleven 3-fold serial dilution steps to accurately determine neutralizing antibody EC_50_ from an EC_50_ titer of 32 (1:8 initial serum dilution x2 for Z-RVP addition x2 for cell addition) to and EC_50_ titer of 100,000 (upper limit of detection). Above an EC_50_ titer of 100,000 (or 5.0 log_10_), a lack of upper asymptote data points led to an increase in variability in the EC_50_ titer. Testing of sera with a potency > 5.0 log_10_ required an additional predilution step of the serum (e.g. start the serum input at 1:200 rather than 1:8).

### Z-RVP-384 assay performance

Several assay parameters were examined to determine the reliability of the assay in determining ZIKV neutralizing antibody titers in sera from human, rhesus macaque, and mouse using the principles and recommendations of the ICH Quality guidelines Q2 Analytical Validation [36]. We determined dilutional linearity, limits of detection and quantitation, repeatability, intermediate precision, and specificity.

#### Dilutional linearity and identification of serum matrix effect

The linearity of an assay is the capability to generate results directly proportional to the expected quantity of analyte in the sample. ICH recommends in its guidelines that the linearity of the assay is evaluated across its range with a minimum of five concentrations tested [36].

**Fig 4:**
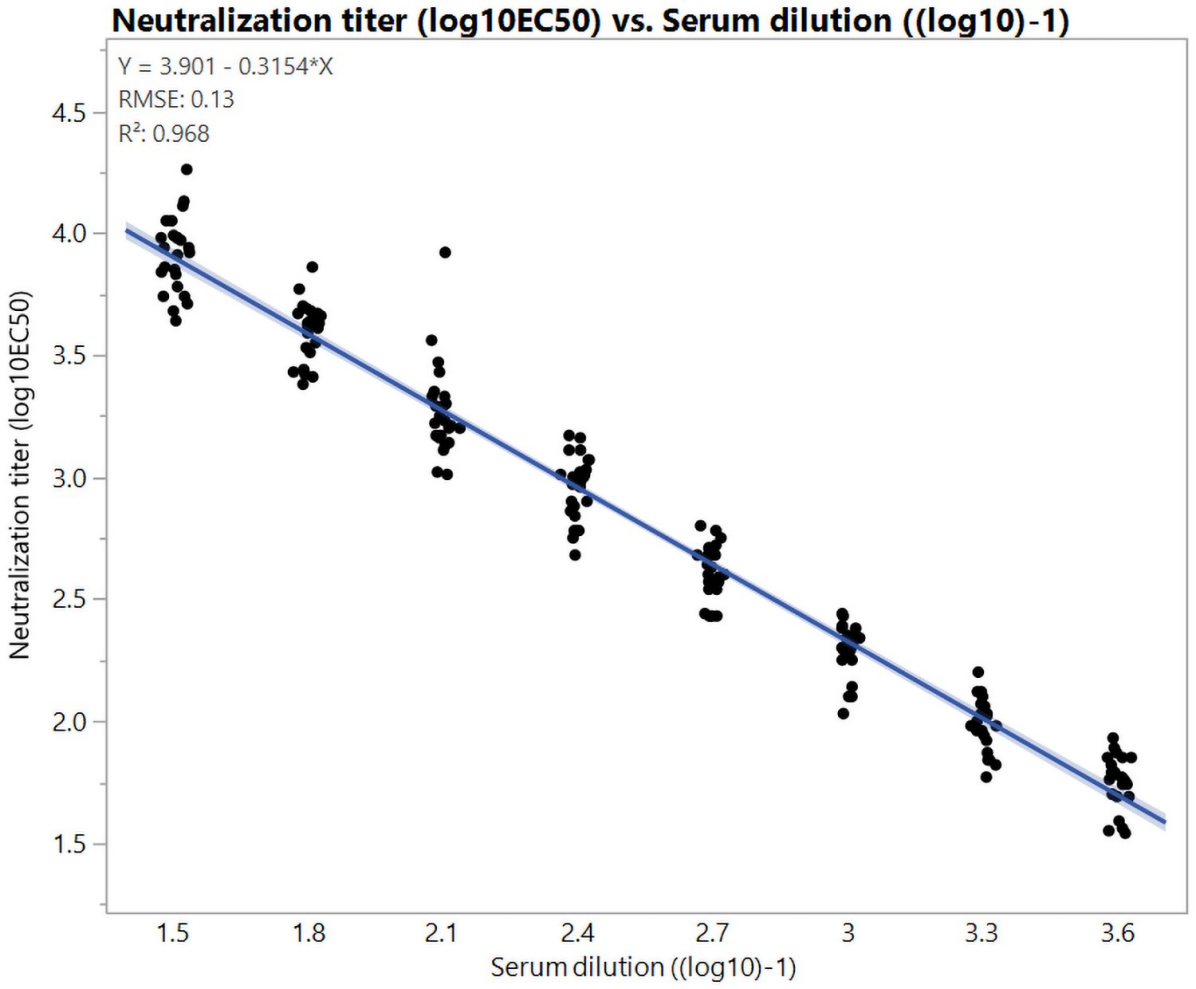
Relationship between serum dilution in assay media and neutralization activity. To examine dilutional linearity in assay media, serum samples were prediluted, starting at 1:8 followed by 2-fold dilutions to generate eight dilutions of samples. The log_10_EC_50_ titers were determined by the RLU signal plotted against the log_10_-transformed serum dilution factor and then fitted with a non-linear regression curve with the lower asymptote constrained to 0 using GraphPad PRISM using RLU’s and lower asymptote constrained to 0. The log_10_EC_50_ values from each dilution were determined and plotted against the input dilutions (log_10_) of the serum. Shown is the best fit line and 95% confidence interval (CI) (y = 3.901–0.3154x, with root mean square error = 0.13, and R^2^ = 0.968 using JMP13.

Dilutional linearity in assay media was evaluated on four occasions using a rabbit ZIKV hyperimmune serum. The serum was diluted in a series of two-fold steps in assay media (1:32 to 1:4096). Each serum dilution was titered six separate times on 4 occasions, generating 24 measurements per dilution resulting in average log_10_EC_50_ neutralizing antibody titers that ranged from 1.54 to 4.26. The assay was linear in assay media over the range tested with R^2^ = 0.968, root mean square error (RMSE) = 0.13 log_10_ (Fig 4). The assay media dilutional linearity was subsequently used to determine the ULOQ.

**Fig 5:**
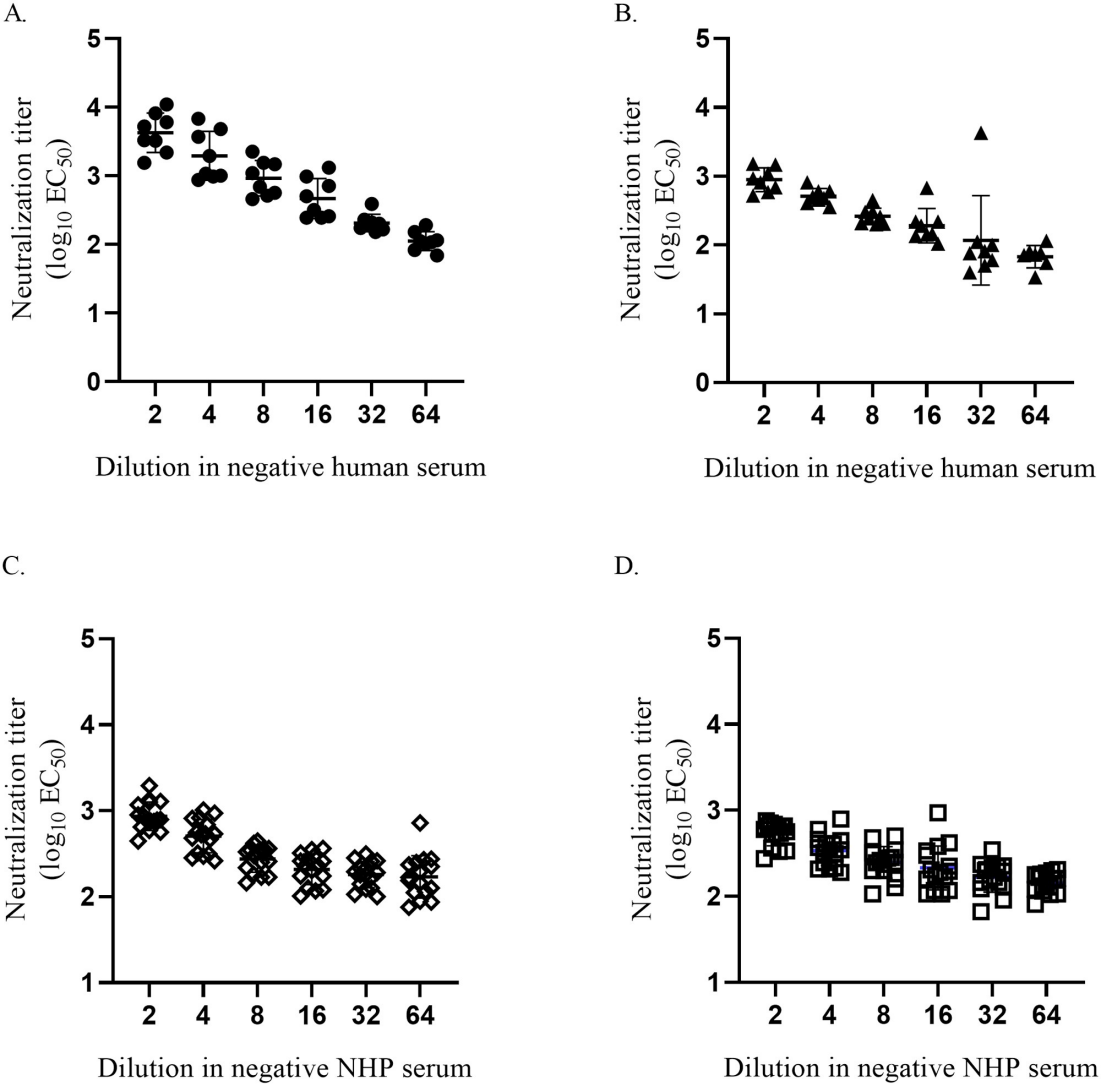
Dilutional linearity of the Z-RVP-384 assay in serum matrix. **(A, B)** Two positive human sera were diluted in negative human serum. **(C, D**) Two positive cynomolgus macaque sera were diluted in negative cynomolgus macaque serum. Serial dilutions test stocks were generated and titrated 8–16 times.

Following dilutional linearity assessment in assay media, a linearity study was utilized to define the matrix effect of the assay in human and cynomolgus macaque serum naïve serum. Two ZIKV antibody positive cynomolgus macaque sera and two ZIKV antibody positive human sera were serially diluted in two-fold steps in negative human serum or cynomolgus macaque serum respectively. Each diluted serum sample was analyzed for log_10_EC_50_ 8 or 16 times (human, cynomolgus macaque samples, respectively) and plotted against the dilution factors (Fig 5).

The assay was linear and accurate in human serum over a serum dilution range resulting in neutralizing titers between ~3.5 log_10_EC_50_ to ~2 log_10_EC_50_ (Fig 5A) and ~3 log_10_EC_50_ to ~2 log_10_EC_50_ (Fig 5B, Table 2). In cynomolgus macaque serum the linear dilution range resulted in neutralization titers of ~3 log_10_EC_50_ to ~2.3 log_10_EC_50_ (Fig 5C) and ~2.8 log_10_EC_50_ to ~2.3 log_10_EC_50_ (Fig 5D, Table 2). The observed neutralization titers determined by serial dilution in serum are summarized as the geometric mean $\sqrt[{n}]{\prod_{i=1}^{n}x_{i}\;}$ of the EC_50_ for each dilution series and compared to expected neutralization titers [37] (Table 2). The expected neutralization titers were calculated using the first determined EC_50_ titer (geometric mean) as reference and dividing by 2 for each 2-fold dilution step (Table 2). A matrix effect was observed for both cynomolgus macaque sera as an increase of observed geometric mean EC_50_ over expected EC_50_ greater than 200% and thereby exceeding expected assay variation of up to two fold. A matrix effect was observed for human serum sample 2 due to a lower starting titer. These results indicated a serum matrix effect, defined as a titer resulting not from ZIKV-neutralizing antibodies but from nonspecific ZIKV inhibitory activity in serum. These detected levels of matrix effect in the four dilution series (Fig 5, Table 2) were the result of the matrix effects of the individual sera (presumed negative) used for the dilution series. Therefore, a serum matrix effect was demonstrated using sera of animals and humans unexposed to flaviviruses.

**Table 2:** Matrix effect observed in human serum and cynomolgus macaque serum.

	**Human serum 1**						**Human serum 2**					
Dilution	2	4	8	16	32	64	2	4	8	16	32	64
observed EC_50_	4132	1883	899	452	203	110	882	509	260	186	101	67
expected EC_50_	reference	2066	1033	516	258	129	reference	441	221	110	55	28
observed % of expected	100	91	87	88	79	85	100	115	118	169	182	242
	**Cynomolgous macaque serum 1**						**Cynomolgous macaque serum 2**					
Dilution	2	4	8	16	32	64	2	4	8	16	32	64
observed geomean EC_50_	834	484	265	201	175	163	513	326	239	204	161	140
expected EC_50_	reference	417	208	104	52	26	reference	257	128	64	32	16
observed % of expected	100	116	127	193	336	626	100	127	186	318	503	871

Final dilutions in serum are shown. Observed % of expected was calculated by observed geomean EC_50_/ expected EC_50_ *100%.

**Fig 6:**
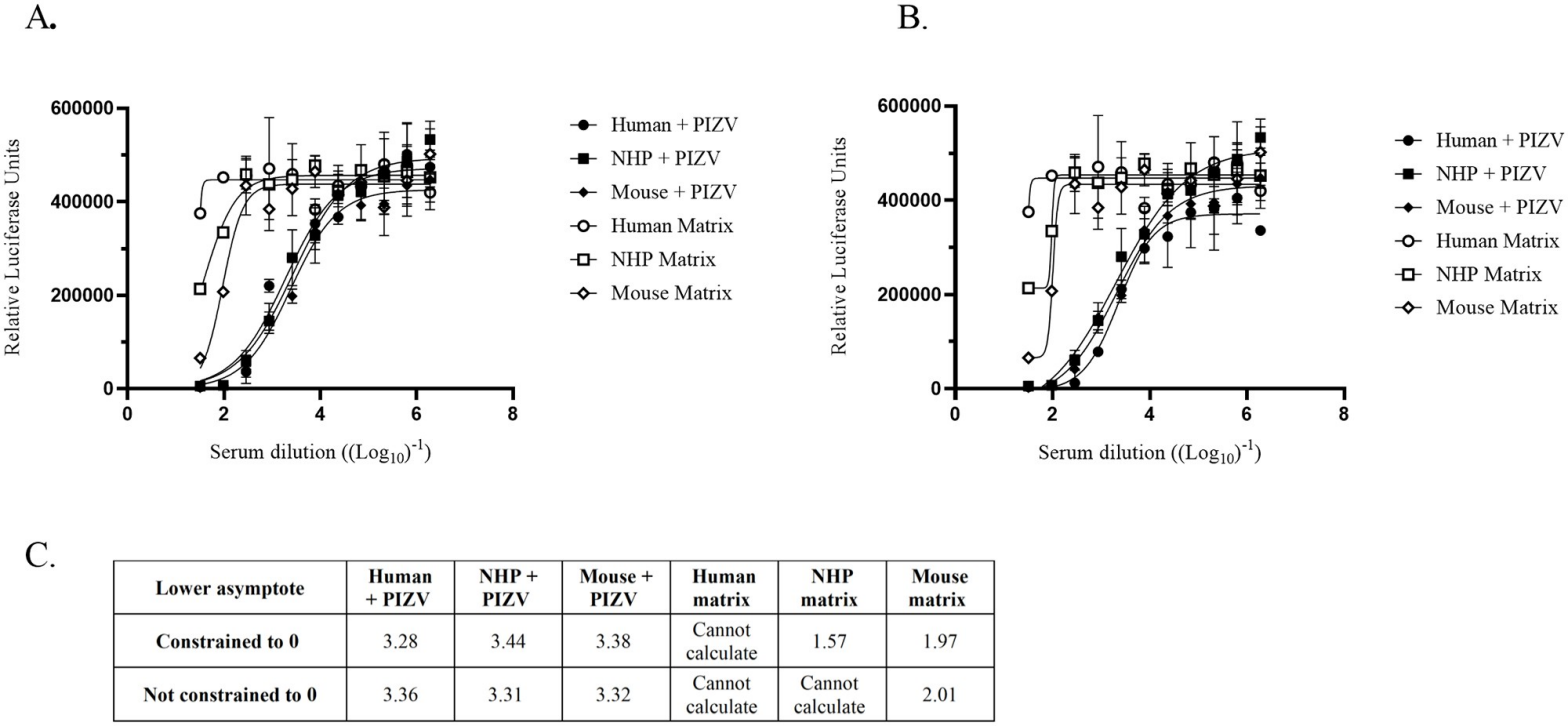
Typical Z-RVP-384 assay neutralization curves of three species using negative and positive sera. Nonlinear regression neutralization curves of human, rhesus macaque, and mouse serum from vaccinated subjects (+ PIZV) or nonvaccinated subjects (Matrix; defined as naïve serum of the species). Each serum dilution was measured in duplicate and the lower asymptote of the nonlinear regression curve was constrained to 0 **(A)** or left unconstrained **(B)**. Neutralization Titers in log_10_ are shown in **(C)** as calculated by nonlinear regression in GraphPad Prism. Cannot calculate = effect on curve was too low to calculate a titer.

Next, we investigated the matrix effect of serum from CD-1 mice and Indian rhesus macaques [38]. Typical regression curves of negative sera which indicate a matrix effect only and of positive sera are shown in Fig 6A and 6B. The ZIKV positive sera were collected from humans, rhesus macaques and CD-1 mice post-immunization with an experimental purified inactivated Zika vaccine (PIZV). In some instances, an EC50 values could not be calculated for negative sera using nonlinear regression curves. Once the nonlinear regression curve’s lower asymptote was constrained to 0 the log_10_EC_50_ titers for negative sera could be calculated while positive sera were unaffected (Fig 6C).

**Fig 7:**
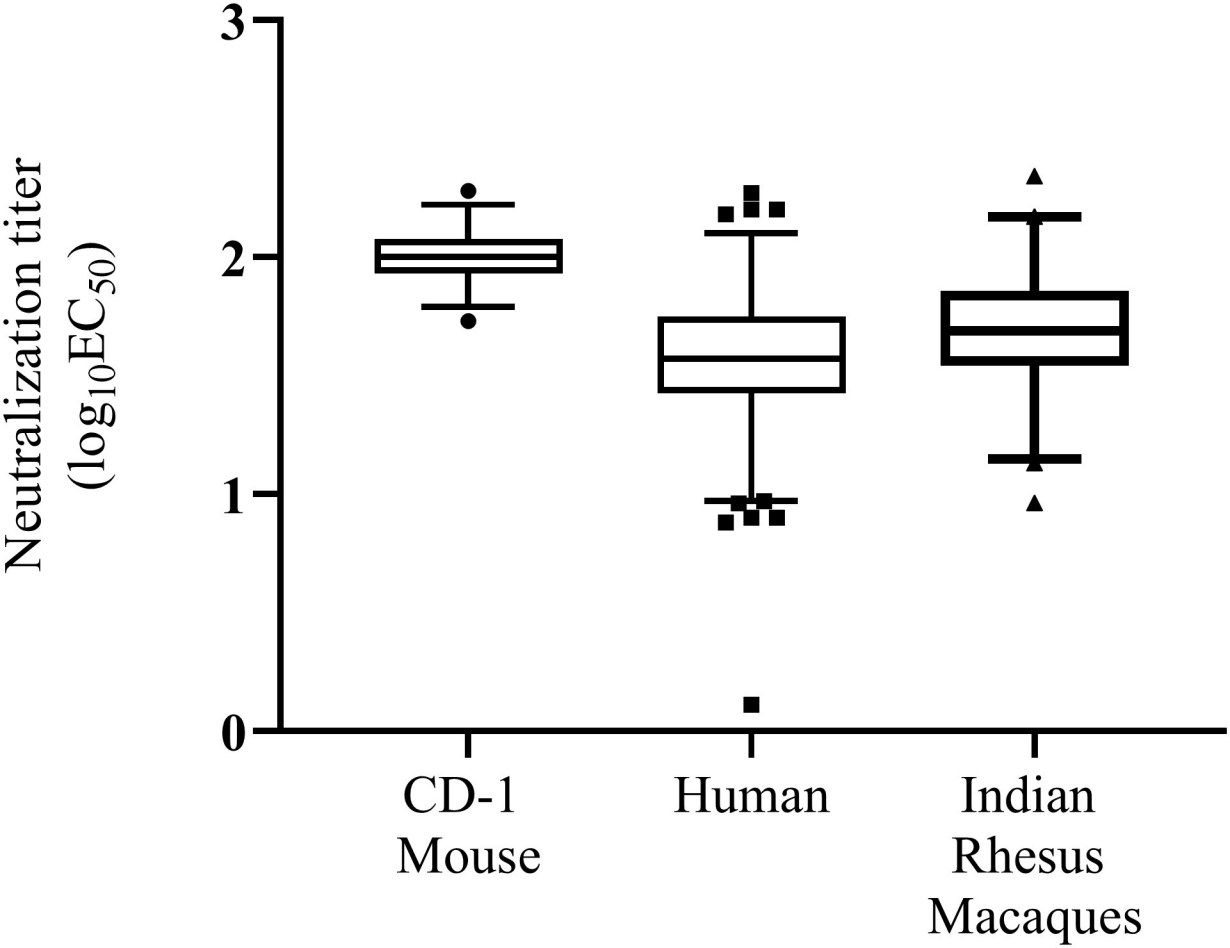
Matrix effect on the Z-RVP-384 assay by species. Quantile plots of log_10_EC_50_ values are shown for 124 human, 84 rhesus macaque, and 69 CD-1 mouse sera. Titers below 1.5 log_10_ are extrapolated (only included for demonstration), and should be reported as < LOD/LLOQ.

To comprehensively evaluate the matrix effect of the serum in the Z-RVP-384 assay, we tested flavivirus-negative sera from human, rhesus macaques, and CD-1 mice. Negativity was determined by lack of flavivirus-binding antibody (a broadly cross reactive flavivirus IgG detection assay) for human and rhesus macaque sera; mouse sera were assumed to be negative. Sera from 124 flavivirus antibody negative humans, 84 rhesus macaques, and 69 CD-1 mice were tested. The upper 95% quantile of the population distribution of negative sera was used to define the threshold between negative sera (showing matrix effect) and positive sera. The 95% quantile for negative human serum was found to be 2.02 log_10_EC_50_, for negative rhesus macaque serum 2.12 log_10_EC_50_, and for CD-1 mouse serum 2.18 log_10_EC_50_ (Fig 7 and Table 3). We assessed whether the small difference between the thresholds for mouse, rhesus macaque and human sera were significant using One-Way ANOVA of means with Tukey’s Honest Difference multiple comparison. The matrix effect of CD-1 mouse serum was significantly higher than both human and rhesus macaque sera (p < 0.0001 for both) and the matrix effect of rhesus macaque serum was higher than that of human serum (P = 0.0002).

**Table 3:** Positive serum cut-off for the Z-RVP-384 assay in human, rhesus macaque and mouse serum.

Species of serum	Sample test number	95% quantile (log_10_EC_50_)
**Human**	241*	2.02
**Rhesus macaque**	84	2.12
**CD-1 mouse**	69	2.18

* includes replicate titration of clinical trial samples.

#### Repeatability and intermediate precision

Assay precision is a measure of the closeness of repeated measurements and can be assessed as repeatability (repeat measurements of a sample at the same occasion with one operator) and intermediate precision (repeat measurements of a sample on several occasions by several operators) [36]. Following ICH guidelines, the robustness of the Z-RVP-384 assay was assessed using the dataset of dilutional linearity in assay media with JMP13 (SAS, USA). The precision was determined by fitting a mixed effects model to the data, with the dilution being a fixed effect and operator, day, and plate being random effects. From this analysis, the intermediate precision of the assay was determined to be 0.14 log10 (Table 4), corresponding to 1.38 fold variability and 38% GCV (geometric coefficient of variation) with manual pipetting. The greatest contributor to assay variance was Day (occasion) confounded by operator, accounting for 57% of the total variance observed (Table 4). A factor contributing to this variance may be differences in Vero cell preparation on a day to day basis (Table 4). Assay repeatability was described by the term ‘within’ and had a SD of 0.08 log_10_EC_50_.

**Table 4:** Variance components of assay robustness assessment.

Component	Variance component	%	SD *
Operator	0.000	0	0.00
Day [Operator]	0.011	57	0.11
Plate [Operator, Day]	0.002	10	0.04
Within	0.007	33	0.08
Total	0.0200	100	0.14

* Standard deviation (SD) calculated as the square root of the variance component

**The term “within” specifies the repeatability of the assay

#### Limits of quantification

**Fig 8:**
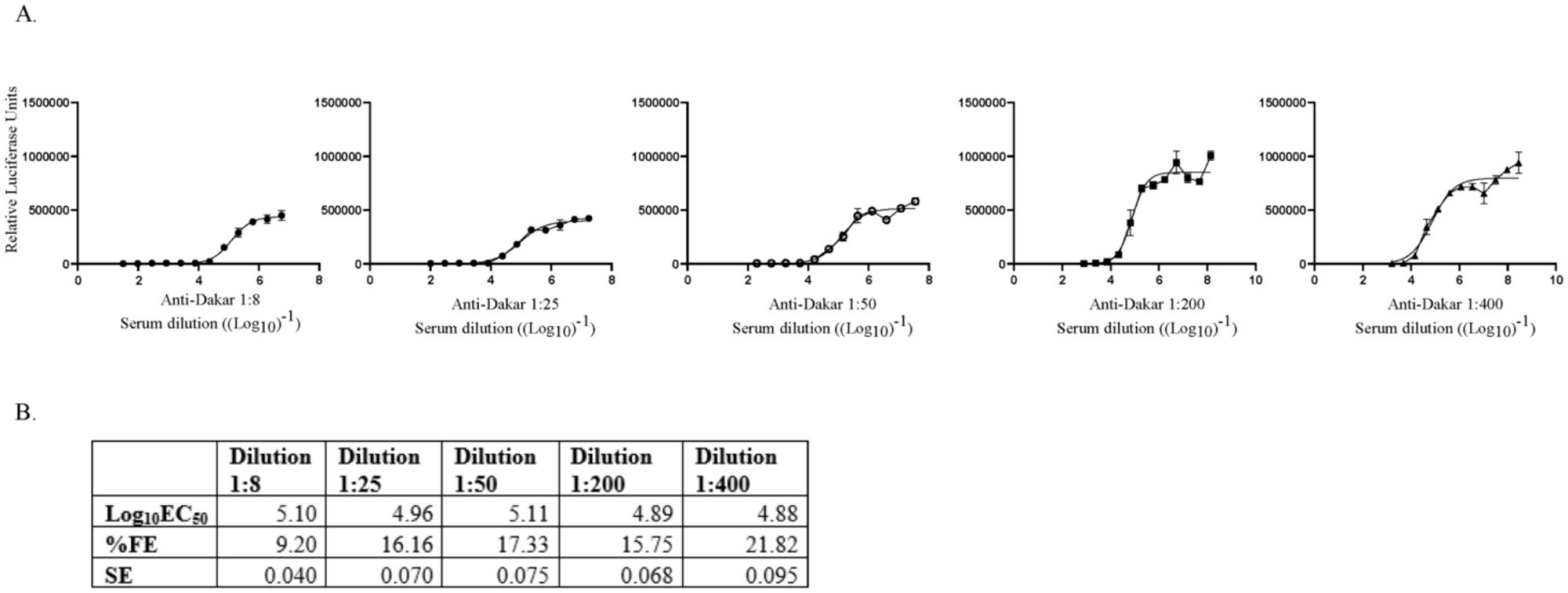
Determination of the ULOQ of Z-RVP-384 assay. **(A)** Neutralization curves of several dilutions of high potency rabbit serum in assay media to estimate the ULOQ for the standard assay. A rabbit hyperimmune serum generated by immunization with live ZIKV (Dakar) was diluted in assay media 1:8, 1:25, 1:50, 1:200, and 1:400. **(B**) log_10_EC_50_ titers generated by each serum dilution shown with Standard error (SE) of the log_10_EC_50_ estimate and % FE (SE*LN(10)*100).

Limits of quantitation are characterized by the upper and lower limits that are both accurate and precise. To estimate the upper limit of quantitation (ULOQ) of the Z-RVP-384 assay, a high titer rabbit hyperimmune serum (estimated titer of ~5 log_10_EC_50_) was pre-diluted 1:8, 1:25, 1:50, 1:200 and 1:400 in assay medium and titered in separate experiments (Fig 8A) the standard error and % Fit Error (FE) was stable from the 1:25–1:200 dilutions (Fig 8B). The upper limit of quantitation (ULOQ) of the Z-RVP-384 assay was dependent on determination of the upper asymptote of the non-linear regression curve (no neutralization, maximum RLU). For highly potent sera few, if any, measurements are made at the upper asymptote, increasing variability in the curve fit and of the EC_50_ determination. No higher potency serum was available for further determination of the ULOQ. A preliminary upper limit of quantitation (ULOQ) for the assay was set at 5.00 log_10_EC_50_ with higher titers requiring retesting of the sample at a pre-dilution of 1:200 in assay media.

To identify the lower limit of quantitation, the precision analysis from the assay media dilutional linearity study and the observed matrix effect were evaluated. The assay was precise to the 1:32 dilution. However, in serum matrix the assay was shown to be linear and accurate down to the matrix effect as described previously.

The Z-RVP-384 assay demonstrated linearity and precision in assay media (Fig 4) beyond the determined serum matrix effect. The matrix effect serves as the LLOQ. Analyte sensitivity is defined by the LLOQ, as such the sensitivity of the assay is restricted to analyte values at or above the evaluated matrix effect. The standard assay range was defined as the interval between LLOQ and ULOQ [36] and resulted in: 105 to 100,000 EC_50_ (human sera), 132 to 100,000 EC_50_ (rhesus macaque sera), and 151 to 100,000 EC_50_ (mouse sera). For sera with potency greater than a 100,000 EC_50_, a predilution can be included for the serum to meet sample acceptance criteria.

#### Neutralizing antibody titers are not affected by species

**Fig 9:**
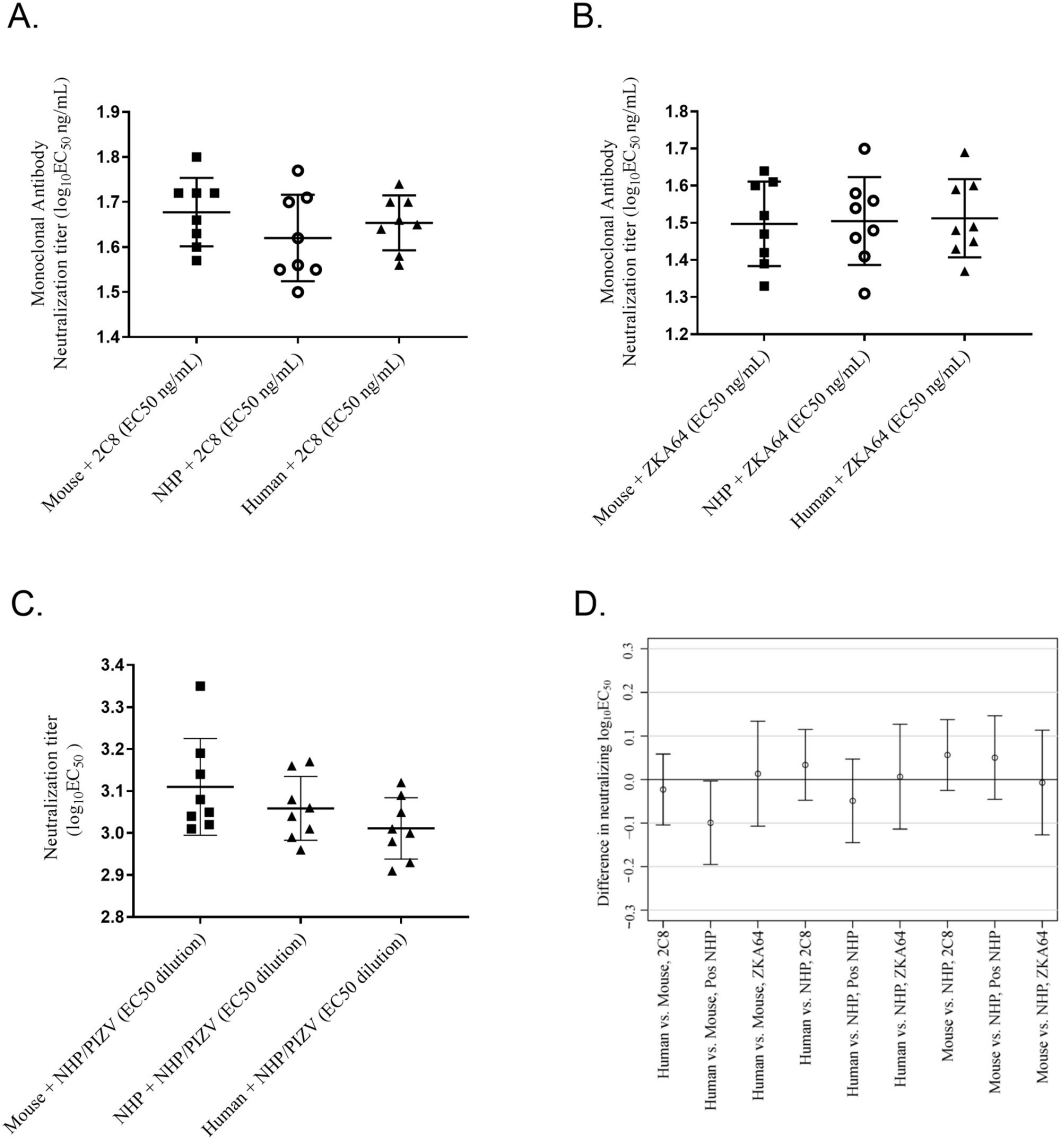
The Z-RVP-384 assay consistently measure neutralizing titers in human, rhesus macaque and CD-1 mouse sera. ZIKV neutralizing titers in human, rhesus macaques (NHP) and mouse sera was measured after addition of **(A)** mAb 2C8; **(B)** mAb ZKA64; **(C)** ZIKV neutralizing rhesus macaque polyclonal serum. **(D)** differences in mean log_10_EC_50_ titers plus 95% CI between the different sera, by spiking group. For mAbs EC_50_ are expressed as ng/mL, and for sera EC_50_ are expressed as reciprocal serum dilution.

Having demonstrated a serum matrix effect of the Z-RVP-384 assay in three species (Fig 7), we investigated whether ZIKV neutralizing antibodies would be measured equivalently by the Z-RVP-384 assay between all three matrices. Negative human, rhesus macaque, and mouse sera were spiked with either monoclonal antibody (mAb) 2C8 or mAb ZKA64, both of which neutralize ZIKV, at a starting mAb concentration of 1 μg/mL. A ZIKV neutralizing rhesus macaque polyclonal serum was also diluted into each negative serum matrix at 1:20. Non-neutralizing mAb 4G2 and negative serum were spiked into the sera as controls. Neutralization titers were determined for each spiked sample eight times (Fig 9A–9C). The absolute neutralization titer values obtained for human, rhesus macaque, or mouse sera spiked with mAb 2C8 or ZKA64 were consistent with previously published observations [39, 40]. No significant differences in neutralization titers were detected among the different serum matrices spiked with mAbs by One-Way ANOVA of means (Fig 9A and 9B). Neutralization titers of sera spiked with the rhesus macaque polyclonal serum were also consistent across the three species with no significant differences detected by one-way ANOVA of means (Fig 9C). A Grubbs test (alpha = 0.05) conducted in GraphPad PRISM identified the result from one mouse serum spiked with macaque serum as an outlier, but inclusion or exclusion of this outlier did not affect the result of the statistical analysis. The differences between neutralization titers of each matrix/spiking group were plotted with 95% CI (Fig 9D). The differences in mean neutralization titers, including all 95% CI fell within the assay variation of 0.2 log_10_ (Fig 9D).

#### Specificity of the Z-RVP-384 assay

Assay specificity is the ability of an assay to assess the analyte in the expected matrix [36]. To determine the specificity of the Z-RVP-384 assay for ZIKV neutralizing antibodies, we tested sera from rhesus macaques vaccinated with ZIKV vaccine candidate PIZV (Takeda, Japan), YFV vaccine (STAMARIL^®^, Sanofi, France), JEV vaccine (IXIARO^®^, Valneva, Austria), WNV vaccine (West Nile -Innovator^®^, Fort Dodge Animal Health, USA), or TBE vaccine (ENCEPUR^®^, GSK, Switzerland). PIZV elicited high neutralizing antibody titers 30 days and 90 days after two immunizations. Other than ZIKV vaccine (PIZV), two of four tested flavivirus vaccines elicited low-level cross-reactive neutralizing antibodies in the Z-RVP-384 assay 30 days after boost vaccination, which dropped to serum background levels by 90 days after second immunization (Table 5). These data demonstrate specificity of the Z-RVP-384 assay for neutralizing antibodies elicited by the candidate ZIKV vaccine when compared to YFV, JEV, WNV, and TBE vaccines.

**Table 5:** Titer in Z-RVP-384 assay of non-human primate sera post-immunization with five different flavivirus vaccines.

Flavivirus/Vaccine name	Mean Log_10_EC_50_ titer^1^	
	30 days post first immunization for YFV and post-second immunization for PIZV, JEV, WNV, TBE	90 days post first immunization for YFV and post-second immunization for PIZV, JEV, WNV, TBE
ZIKV vaccine (PIZV^2^)	4.62 (SD: 0.14)	4.10 (SD: 0.06)
YFV/ STAMARIL^®^	<LLOQ	< LLOQ
JEV/ IXIARO^®^	2.13 (SD: 0.11)	< LLOQ
WNV/ West Nile -Innovator^®^	2.20 (SD: 0.30)	< LLOQ
TBE/ ENCEPUR^®^	<LLOQ	< LLOQ

^1^ Mean titers of sera from 4 macaques per flavivirus vaccine group

^2^ Candidate ZIKV vaccine

LLOQ of the assay is 2.12 Log_10_EC_50_

### Correlation of Z-RVP-384 and PRNT assay

**Fig 10:**
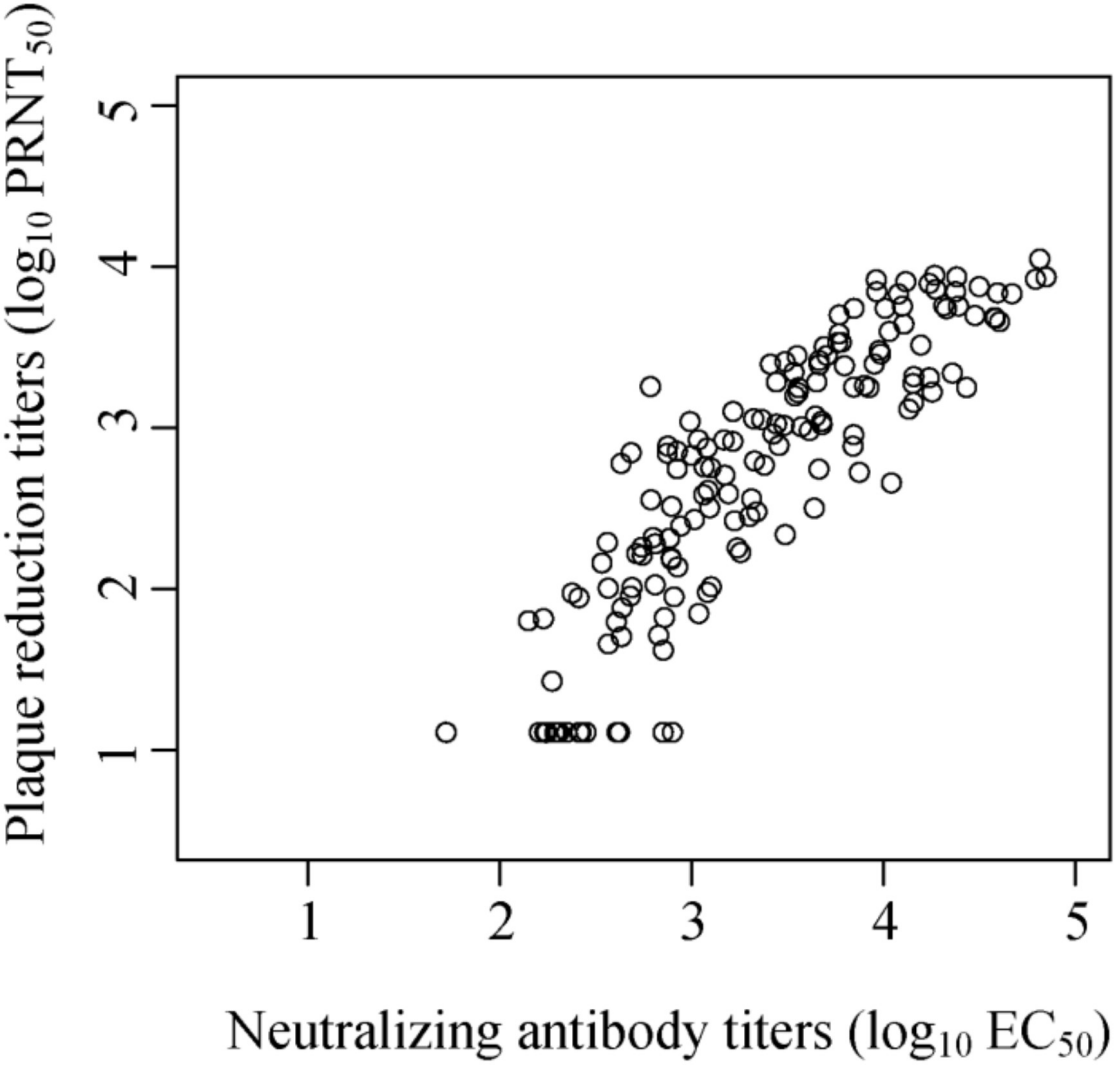
Correlation of ZIKV neutralization titers in human sera using Z-PRNT and Z-RVP-384 assays. Human sera from a clinical trial of PIZV (ZIK-101) day 29 (post dose one sera) and day 57 (post dose two sera) were tested in both Z-PRNT (Y axis) and Z-RVP-384 (X-axis) and log_10_-transformed titers plotted. A Spearman correlation analysis was conducted in R and showed a strong correlation of 0.94.

We performed a correlation analysis of the Z-RVP-384 assay with a ZIKV PRNT assay (Q2 Solutions, San Juan Capistrano, CA). The comparison used serum titer results from over 120 subjects collected after first (day 29) and second PIZV vaccinations (day 57) from clinical trial ZIK-101 (ClinicalTrials.gov identifier: NCT03343626) [41]. For correlation analysis, Z-RVP-384 titers and PRNT titers below their respective lower limits of quantitation (LLOQ) were replaced with half of the LLOQ value. Correlation of results between the two assays was highly significant with a Spearman correlation coefficient of 0.94 (Fig 10). This suggests that the Z-RVP-384 and PRNT assays are measuring the same or similar antibody-virus interactions.

### Performance of the international ZIKV antibody standard in the Z-RVP-384 assay

Assay accuracy compares the value determined by an assay to an accepted value such as that assigned to a reference standard [36]. No reference standard for ZIKV neutralizing antibodies is available. However, an international serum standard (16/352) for ZIKV antibodies has been developed by WHO and NIBSC which has been assigned an international unit value (250 IU/ampoule) [42]. A prototype of the Z-RVP-384 assay was used in the evaluation of this standard [42]. We retested the international serum standard (16/352) in the updated Z-RVP-384 assay. NIBSC standard 16/352 was reconstituted and aliquoted into single use aliquots for testing by 4 different operators on 9 separate occasions in the manual Z-RVP-384 assay. By Z-RVP-384 the standard 16/352 had an average titer of 4.24 log_10_EC_50_ and Standard Deviation of 0.2 log_10_EC50.

### Automation of serum preparation

An important element of ZIKV neutralizing antibody assay development was capability for high throughput to enable rapid testing of large numbers of clinical samples. A proof of concept assessment of Z-RVP-384 assay automation was conducted to determine feasibility of a high throughput operation. As an initial step, serum serial dilution was automated using a Tecan 780 Fluent liquid handler using a 384-tip head (Tecan, Switzerland). Two positive controls, one negative control and 11 serum samples ranging in ZIKV neutralization titers from negative to high positive were run once per plate on 6 plates per occasion (day) and on 4 separate occasions to generate a total of 24 measurements per sample. The location of the samples on each plate was varied among the 6 plates to maximize assay variation. In total 336 measurements were performed.

Five samples failed the sample acceptance criteria, a 1.49% failure rate. Using automation, the assay GCV of the log_10_EC_50_ titers was 22%, compared with 38% GCV of the manual Z-RVP-384 assay and time for the serum dilution step was reduced by 50%.

## Discussion

ZIKV caused a significant epidemic in South America, Central America, and the Caribbean during 2015–2016 with 1 million cases, resulting in the declaration of a Public Health Emergency of International Concern by WHO [43, 44]. As response to this outbreak, multiple efforts have been initiated to develop a safe and effective vaccine with several candidates currently in clinical development [45, 46]. Neutralizing antibodies against ZIKV have been proposed as an important immune marker for clinical benefit [12, 14–16, 47]. Several assays have been developed to measure the level of ZIKV neutralizing antibodies in serum. The traditional flavivirus neutralization assays, the PRNT and MNT assays, are low throughput, imprecise, manual labor intensive and represents a major bottleneck in testing large numbers of sera for clinical studies [18, 19, 21]. To address some of the limitations of the traditional PRNT and MNT assays, we have developed a reproducible, high throughput, automatable 384-well plate ZIKV RVP assay for measuring serum antibodies in humans, rhesus macaques and mice, and that eliminates use of infectious ZIKV.

Immunogenicity, efficacy and safety in nonclinical models is an important aspect of development of ZIKV vaccine candidates [48, 49] as the current low prevalence of ZIKV infections make Phase III efficacy studies difficult to conduct. To facilitate comparison, and potentially bridging of immunogenicity data from animal models to humans the ideal assay would use the same reagents regardless of species, and titer measurement should be unaffected by serum matrix from the different species [48–50]. We detected a matrix effect in unvaccinated (naïve) animals and humans with our Z-RVP-384 assay and used a population approach to set a cutoff value between naïve sera and sera containing ZIKV neutralizing antibodies. As demonstrated by spiking experiments, the serum matrix from the different species did not affect RVP titers above the cutoff value indicating that the Z-RVP-384 assay can be used to compare neutralizing antibody titers across different species. A low level of flavivirus cross-reactive antibodies could be detected in the Z-RVP-384 assay. The effect of prior flavivirus exposure on ZIKV infection or ZIKV vaccination is not well understood and therefore further studies are required. The development of a ZIKV specific neutralizing antibody assay to evaluate ZIKV vaccine efficacy and to determine a surrogate endpoint that predicts clinical benefit would be of great value in an flavivirus endemic area.

We observed a strong correlation between neutralizing titers from the Z-RVP-384 assay and the PRNT assay, suggesting that the two assays are measuring the same antibody-virus interaction including blocking cell entry. Others have also explored other assays for measuring ZIKV neutralizing antibodies such as RVP (WNV-based replicon) neutralization assay, using a flow cytometer and a GFP readout [29], RVP (WNV-based replicon)-based microneutralization assay, using a 96-well format and a GFP readout [28], RVP (DENV-based replicon) based microneutralization assay using a 96-well format and chemiluminescence readout [16]. Multiple parameters of flavivirus neutralizing antibody assays vary among labs, complicating study comparisons. The use of international standards, such as the WHO/NIBSC standard 16/352 [42] could be of great value to compare absolute titer results determined by different assays, on different sample sets, and by different laboratories.

## Conclusions

We have developed a safe, robust and automatable ZIKV RVP neutralization assay that reproducibly measures neutralizing antibody titers in sera from multiple species, correlates with a qualified ZIKV PRNT assay, relies on commercially available reagents and is suitable for validation according to 21 CFR part 11. This Z-RVP-384 assay lends itself to the reliable assessment of large sample sets for clinical trials.

## Material and methods

### Ethics statement

The sera used for Z-RVP-384 assay development were obtained from animal studies that were approved and conducted in strict accordance with the Guide for the Care and Use of Laboratory Animals of the National Research Council. Details of the studies are described in the S1 File.

#### CD-1 mouse study

All in-life practices, including husbandry and environmental enrichment, were approved and conducted per Institutional Animal Care and Use Committee (IACUC) of Millennium Pharmaceuticals, Inc. Cambridge, MA USA under protocol #16-06-175. Euthanasia (carbon dioxide asphyxiation) was performed in accordance with accepted America Veterinary Medical Association (AVMA) guideline.

#### Rabbit study

All in-life practices, including husbandry and environmental enrichment, were approved and conducted per IACUC of Covance under protocol 0141–16. Rabbits were monitored for signs of morbidity and/or mortality daily according to animal welfare act and AAALAC. Rabbits were given Ketamine and Xylazine (anesthesia and analgesia) before euthanization by CO2 inhalation and exsanguination.

#### Primed rhesus macaque study

All in-life practices, including husbandry and environmental enrichment, were approved and conducted per IACUC of Inotiv under protocol #2384–14376. Macaques were monitored at least twice daily for stress or suffering, and body weight was measured on D1 prior to dosing and monthly thereafter. Cageside observations included observation for mortality, moribundity, general health, and signs of toxicity. Treatment with the vaccines had no effect on mortality, physical examinations, cageside observations, body weights or body weight changes. Moribund animals and all surviving animals at the end of the study were euthanized by intravenous injection of sodium pentobarbital (or equivalent) and exsanguinated.

#### Flavivirus Naïve rhesus macaque study

Rhesus macaque sera were obtained from a previously published PIZV vaccination and ZIKV challenge study [51] conducted at Charles River Laboratories (CRL), Mattawan. MI. All in-life practices, including husbandry and environmental enrichment, were approved and conducted per IACUC of CRL under protocols #2715–001 and #2715–002.

#### Cynomolgus macaque study

Cynomolgus macaque sera were obtained from a previously published ZIKV challenge study conducted at Wisconsin National Primate Research Center [52]. Madison, WI USA. All in-life practices, including husbandry and environmental enrichment, were approved and conducted per IACUC of The University of Wisconsin-Madison, College of Letters and Science and Vice Chancellor for Research and Graduate Education Centers Institutional Animal Care and Use Committee under protocol number G005401-R01.

### ZIKV reporter virus particle (Z-RVP) reagent

Reporter virus particles (RVP) were purchased from Integral Molecular (Philadelphia, PA) ZIKV reporter virus particles (Z-RVPs) contain CprM/E from ZIKV strain SPH2015 packaged within the particle a DENV-based replicon with the *Renilla* luciferase gene [32]. The Z-RVP reagent is replication deficient but is capable of infecting permissive cells to deliver the replicon which drives the expression of *Renilla* luciferase. Addition of an appropriate substrate results in the generation of a chemiluminescent signal, which is measured to determine sample neutralization activity. The SPH2015 strain of ZIKV was the first strain isolated from the outbreak in Brazil in 2015 and has a high degree of genetic similarity to other ZIKV strains from the same outbreak in South and Central America during 2015–16 and other recent Asian-lineage ZIKV strains [53].

Eight RVP lots were used during the development of the assay (lot 135 A-D, 164 A-D). Each lot number represents a different production run, each letter of the lot represents a different harvest date.

### Assay media

Opti-MEM no phenol red (Gibco, 11058–021) supplemented with 10% Fetal Bovine Serum, heat-inactivated (Sigma, F4135-100ML, lot 17L179) and 1% Penicillin/Streptomycin (Gibco, 15140–122).

### Vero cells

Vero cells were obtained from ATCC (CCL-81.4) and expanded into a research cell bank (non-CGMP). The cell bank was confirmed to be mycoplasma negative. Cells were maintained in DMEM Growth media (Corning, 15017CV) plus 10% FBS, (Sigma, F4135-100ML, 17L179), 2mM L-Glutamine (Gibco, 25030–054) in 5% CO_2_, at 37 ± 2°C using tissue culture flasks (Corning, 10-126-061). Vero cells were split with 0.05% EDTA-Trypsin (Gibco, 25300).

### Sera and monoclonal antibodies

All sera were heat-inactivated at 56°C for 30 min. Positive serum was defined as serum that contained ZIKV-neutralizing antibodies tested using a CPE based microneutralization assay. Negative serum was defined as serum that had no neutralizing activity against ZIKV above the species-specific matrix effect. The negative serum used throughout assay development and carried forward as assay acceptance control was negative rabbit serum (IGMS-SER, Innovative Research, USA). The positive serum used throughout assay development was generated by Takeda by immunizing rabbits with live ZIKV preparation using strain Dakar41524 and strain PRVABC59 (anti-Dakar L777P16, anti-Dakar L821P119, anti-PRVABC59 L773P08). These positive sera were used to optimize the assay and as acceptance controls. Test sera to establish species specific matrix cut-offs came from mouse (CD-1 strain), from rhesus macaques, and from humans (clinical trial ZIK101, clinicaltrial.gov identifier: NCT03343626). The rhesus macaque sera and human sera were determined to be negative for flavivirus-binding antibodies by flavirus-screening luminex assay (Luminex^®^ Corp (Austin, TX, USA) and Ampersand Biosciences (Lake Clear, NY, USA).

Two monoclonal antibodies (mAb) were used in the assessment of the Z-RVP-384 assay: ZKA64 is a neutralizing mAb that has been reported to be ZIKV specific [40]. 2C8 is an EDE domain antibody that neutralizes both DENV (serotype 1–4) and ZIKV [54].

### CPE based microneutralization assay

A cytopathic effect (CPE) based neutralization assay was used to determine neutralizing capacity of serum. At the time of the assay, serum and ZIKV aliquots were thawed. Two-fold dilution series of the serum was mixed with ZIKV (200 TCID50) in DMEM 2%FBS. Titers were determined by inoculating 100 μL of the preincubated serum/ZIKV mixture to freshly confluent monolayers of Vero cells grown in 96-well plates. The plates were incubated under 5% CO2 for 5 days at 36°C ± 2°C, before visual observation of the cell monolayer under a microscope for the presence of CPE resulting from viral infection. The neutralization titer was reported as the highest serum dilution that lacked visible CPE.

### *Renilla*-Glo Luciferase assay system and CellTiter-Glo cell toxicity detection system

The *Renilla*-Glo Luciferase assay system (Promega, USA, catalog number E2720) and the CellTiter-Glo detection system (Promega, USA; catalog number G7570) was used per manufacturer’s instructions for chemiluminescent detection. Tissue-culture treated 384-well white opaque bottom plates (Corning, 3570) were used.

Briefly, assay plates were left to come to ambient temperature (room temperature, 20–22°C) for 15 min. Substrate was removed from -20°C and mixed to buffer (stored at room temperature for up to 3 months, or long-term at -20°C) at 1:100 dilution. Substrate/buffer was added to the assay plates in a biosafety cabinet and incubated for 15 min at room temperature in the dark. The plates were read within 60 min.

### Electronic pipettes

These procedures were carried out using Thermo Scientific pipettes, including E1-Cliptip electronic 8 channel of various volumes, E1-Cliptip Equalizer 384 30 with 8 and 12 channels and E1-Cliptip Equalizer 384 125 with 8 channels. All pipettes were used with current software versions and were calibrated yearly. Pipettes of equivalent accuracy and precision can be substituted.

### Chemiluminescence reader

The EnSpire Alpha Multimode Plate Reader (model 2300 or 2390) by Perkin Elmer was used for chemiluminescent detection. Equivalent readers may be used.

### Data conversion to titers

Initially, JMP12 and JMP13 were also used with a custom script to generate 4PL curves. GraphPad Prism version 7 or 8 was used for the log_10_EC_50_ and EC_50_ titer generation during assay development. In brief, serum serial dilutions are transformed to log_10._ Relative luciferase units are plotted against the log_10_ serum dilutions. A non-linear regression curve fit is performed with the lower asymptote constrained to 0.

### Final optimized parameters of the Z-RVP-384 assay

Lab Protocol can be found here dx.doi.org/10.17504/protocols.io.bscpnavn

(1) Sera from human, mouse, rabbit, or non-human primate, are heat-inactivated (HI) at 56°C for 30 min and then serially diluted in assay media. (2) Serially diluted HI-serum is then transferred to a white tissue-culture treated 384-well flat bottom plate in a final volume of 7.5 μL. (3) Z-RVP particles are added (7.5 μL volume and Z-RVP concentration optimized by lot) and (4) the plate is incubated at 37°C for 60 min in a 5% CO_2_ humidified incubator to allow neutralization. (5) After the neutralization step Vero cells are added to the 384-well plates at 4,625 cells per well in 15 μL volume and (6) incubated at 37°C for 72 hrs in a 5% CO_2_ humidified incubator. RVPs that were not neutralized are able to infect cells and produce *Renilla* luciferase. (7) To detect the *Renilla* luciferase activity in the cells, the plates are equilibrated to room temperature for 15 min, and then incubated with *Renilla*-Glo^™^ Luciferase reagent according to manufacturer’s instructions (Promega, Madison, WI). Chemiluminescence is read with an EnSpire chemiluminescence reader (Perkin Elmer, USA) or equivalent as relative luciferase units (RLU), followed by (8) data conversion to neutralization titers (EC_50_ or log_10_EC_50_). Neutralization titer is expressed as inverse of the serum dilution which reduces the relative luciferase activity by 50%. The optimal serum input dilution is 1:8 in assay media for the standard assay and is adjusted 2-fold with the addition of the RVP reagent volume (to 1:16) and a further 2-fold with the addition of the cell suspension to 1:32 as the lowest serum input dilution presented here. To generate Effective Concentration 50% (EC_50_) titers, nonlinear regression curves are fit to relative luciferase units plotted against serum dilution (in log_10_) with the lower asymptote constrained to 0 using GraphPad PRISM (version 7 or higher). The log_10_EC_50_ titers are normally distributed, the EC_50_ titers are not.

### Statistical analyses

Log_10_-transformed EC_50_ titers were used for all statistical tests as they were normally distributed. Group comparisons were carried out using One-Way ANOVA of means with Tukey’s multiple comparison in GraphPad PRISM (version 7 or 8) or JMP13 (SAS, USA).

For the interleaved assay, the mean (AVG), SD and CV (of the mean) for each signal (max, mid, min explained under Signal stability section) on each plate. The CV takes into account the duplicates wells that will be used in the assay ($CV=\frac{\frac{SD}{\sqrt{2}}}{AVG}$) [35]. For the mid-signal wells the % activity relative to the means of the max and mid signals was calculated per plate ($\%Activity=\frac{Well(mid)-AVG(min)}{AVG(max)-AVG(min)}\times 100$) [35]. The mean and SD for the mid-signal percent activity values were calculated per plate. The Signal Window (SW) was calculated for each plate $SW=\frac{\left(AVG(max)-\frac{3SD(max)}{\sqrt{n}})-(AVG(min)+\frac{3SD(min)}{\sqrt{n}}\right)}{\frac{SD(max)}{\sqrt{n}}}$, where n is the number of replicates of the test substance that will be used in the assay (n = 2) [35]. The Z’ factor (Z’) was calculated for each plate using the formular $Z-factor=1-\frac{3(\sigma p\;+\;\sigma n)}{\left|\mu p\;-\mu n\right|}$ [35].

To assess the robustness of the assay, the total variation of the assay was assessed in JMP version 13. To determine which factors have the greatest influence on assay variability a variance component analysis was conducted in JMP (SAS, USA). In the variance component analysis, the SD) was calculated as the square root of each variance component estimate. The % GCV was calculated by the formula $\%\;GCV={100\%*(e}^{\text{ln}\left(10\right)*SD}-1)$ [37].

To assess the level of correlation between Z-RVP-384 and PRNT titers, a Spearman correlation was determined in R version 3.2.2.

### Plate acceptance criteria

We developed several acceptance criteria based on Z-RVP-384 assay performance characterization: required assay signal (RLU) and positive and negative control ranges. Maximum signal (RLU) of the Z-RVP-384 assay must be greater than 100,000 RLU and less than 10,000,000 RLU, the detection limit of the EnSpire Alpha chemiluminescence readers we used (Perkin Elmer technical guidance). Acceptance ranges for the positive and negative serum controls included on each plate were established as mean log_10_EC_50_ ± 3x SD for the positive control and mean log_10_EC_50_ + 2x SD for the negative control. All samples on a failed plate must be retested.

### Sample titer acceptance criteria

The fit of the non-linear dose response curve to the observed data points is assessed by % FE with an upper limit of 40% for all sera above the LLOQ. Every sample that fails one or more titer acceptance criteria is retested. The sample is considered negative for ZIKV-neutralizing antibodies when the log_10_EC_50_ titer is below the species-specific serum matrix cut-off, which also serves as the LLOQ and the LOD (Table 3). The ULOQ for the standard assay (1:8 serum dilution in assay media as input) is 5.00 log_10_EC_50_. Any sample > ULOQ is re-tested using a pre-dilution of 1:200 or greater.

We observed a 1% disagreement rate between accepted titers when measuring 429 samples independently by two operators, indicating that the acceptance criteria contribute to a high level of titer consistency.

### Flavivirus-screening assay

The Flavivirus Screening assay was developed in collaboration with Luminex^®^ Corp (Austin, TX, USA) at Ampersand Biosciences (Lake Clear, NY, USA). Serum samples were diluted 1:2,000-fold in sample dilution buffer (PBS, 0.05% ProClin 300). 30 μL of the diluted sample was transferred to a 96-well plate including negative, low and high positive controls. 10 μL diluted multiplexed serological panel bead mix (1500 beads per antigen) was added to each sample followed by a 60-minute incubation (room temperature, in the dark, orbital shaker 850 rpm). The beads were washed three times with 100 μL of assay buffer (PBS, 0.05% Tween 20, 0.05% ProClin 300). 40 μL Anti-IgG PE was added to each well and incubated 30 min at room temperature with shaking. The beads were washed three times as above and resuspended in 100 μL assay buffer and analyzed using a Luminex^®^ 200^™^. Greater than 35 beads per antigen were read and plates accepted based on control ranges and IgG bead having >20,000 median fluorescent intensity (MFI).

## Supporting information

S1 File Animal study design. (DOCX)

S1 Checklist The ARRIVE guidelines 2.0: Author checklist. (PDF)
