# Supplementary material for: A high throughput reporter virus particle microneutralization assay for quantitation of Zika virus neutralizing antibodies in multiple species
Source: PLoS One. 2021 Apr 23;16(4):e0250516. doi: 10.1371/journal.pone.0250516 (PMC8064526; doi:10.1371/journal.pone.0250516)
Supplement: S1 File — (DOCX) [file pone.0250516.s001.docx]

**S1 File. Animal study design**

**Immunogenicity of PIZV in CD-1 mice.**

All in-life practices, including husbandry and environmental enrichment, were approved and conducted per Institutional Animal Care and Use Committee (IACUC) of Millennium Pharmaceuticals, Inc. Cambridge, MA USA under protocol #16-06-175. Seventy, 4-6 week old CD-1 mice (male and female) were acclimated for three days before start of the study and were randomly assigned to groups by weight-order distribution before dosing. Clinical observations were performed prior to randomization and animals in poor health were not assigned to the study. Sample size and power were determined using priori analysis in SAS JMP Version 13 for each individual group and all groups together. Mice were housed three per cage, males and females separated.

To determine the immunogenicity of PIZV in CD-I mice, seven groups of 10 CD-1 mice were inoculated intramuscularly (IM) with different doses of PIZV or PBS on Day (D)0 and D28. PIZV (CTM1 1µg/100µL with 0.04mg of elemental aluminum as Alhydrogel® (alum)) was diluted with PBS and alum to prepare 0.01 and 0.05 µg PIZV/100µL with 0.04 mg/100µL alum (Groups 1 & 2). PIZV (Eng) was formulated at concentrations of 0.01 µg, 0.05 µg and 1 µg per 100µL doses with 0.04 mg/100µL alum (Groups 4, 5 & 6 respectively). Group 7 was a placebo control with 1X PBS mixed with alum (final concentration of 0.04 mg/100 µL). All PIZV formulations were prepared on their day of use. Serum samples were collected on D-1 (prebleed), D27 and D56 to determine mean neutralizing antibody titers (EC_50_). Data analysis was performed in Graph Pad Prism 7 as described under the statistical analysis section. Treatment groups were compared by 1-way ANOVA and Tukey’s Multiple Comparison in GraphPad Prism. No animals were excluded from data analysis. All animals (n=60) receiving PIZV (CTM1 or Eng) elicited a neutralizing antibody response as compared to placebo (n=10). Serum samples from D-1 (prebleeds) were used for Z-RVP-384 assay LLOQ estimation for CD-1 mice.

Mice were monitored daily for stress or suffering, and body weight (BW) was taken as per protocol. Humane endpoints to euthanize mice included 20% BW loss from relative start of the study, 15% BW loss over a 24hr period, respiratory distress, impaired righting reflex, or moribund condition. Euthanasia (carbon dioxide asphyxiation) was performed in accordance with accepted America Veterinary Medical Association (AVMA) guidelines.

**Polyclonal Antibody Production against 2 Strains of Zika Virus in Rabbits**

All in-life practices, including husbandry and environmental enrichment, were approved and conducted per IACUC of Covance under protocol 0141-16. Eight, 7 month old female New Zealand White (NZW) rabbits were acclimated for 5 days before start of the study and were arbitrarily assigned to groups before dosing. Rabbits were housed one per cage. The number of rabbits was based on the volume of pooled sera required to be used as positive control sera for serological assay development and for ZIKV neutralization during PIZV process development. Two groups of 4 NZW rabbits were immunized subcutaneously with 1.58 x 10^9^ PFU of ZIKV DakAr 41524 strain (Group 1) or with 3.16 x 10^8^ PFU of ZIKV PRVABC59 strain (Group 2) on D0, D28, D56 D84, and D113. Serum samples were collected prior to immunization on D0, D28, D56, D84, D113, D140, D168 and D196 and ZIKV neutralizing antibody titers were determined using a CPE-based microneutralization assay. Prebleeds were used as negative control sera. High neutralizing antibody serum was pooled and used as positive control sera for serological assay development. Specifically, D28 serum samples pooled from each group and D56, D84 and D113 serum samples pooled from Group 1 were used as positive controls for Z-RVP-384 assay development. Statistical analysis was not required for this study and no animals were excluded from analysis. Rabbits were monitored for signs of morbidity and/or mortality daily according to animal welfare act and AAALAC. Rabbits were given Ketamine and Xylazine (anesthesia and analgesia) before euthanization by CO_2_ inhalation and exsanguination.

**Flavivirus primed rhesus macaques study**

All in-life practices, including husbandry and environmental enrichment, were approved and conducted per IACUC of Inotiv under protocol #2384-14376. Macaques were screened for antibodies against flavivirus (ZIKV, DENV, YFV, JEV, WNV, Usutu, Saint Louis Encephalitis (SLE)), and alphavirus (Chikungunya virus using the flavivirus screening assay. The macaques tested negative for Herpes B Virus, Simian Retrovirus, Simian Immunodeficiency Virus, Simian T Lymphotropic Virus, Mycobacterium Tuberculosis, Simian Varicella Virus, Malaria, Salmonella, Shigella, Yersinia and internal parasites.

Twenty flavivirus negative male macaques (ranging from 2-3 years old and weighing 3-6 kg) were acclimated for 31 days before prior to the first vaccine dose. Sample size and power were determined using priori analysis based on two samples, two sided t-test with p-value threshold of 0.05. Animals underwent physical examination prior to manually being assigned to study groups based on weight to achieve a similar average weight per group. Following group assignment, each study animal was assigned a unique number. Animals were partnered within the same sex and group.

To determine the immunogenicity of PIZV in flavivirus primed macaques, five groups of 4 rhesus macaques were inoculated intramuscularly (IM) with a single dose of YFV (Group 1) on D1 or two doses of PIZV (Group 2), JEV (Group 3), WNV (Group 4) or TBE (Group 5) on D1 and D29. The commercial vaccines (YFV, JEV, TBEV and WNV) were administered according to the manufacturer’s instructions. PIZV was dosed as 10 µg/0.5mL with 0.4 mg of alum). PIZV was administered IM to Group 1on D169 and D197 and to Groups 3, 4 and 5 on D197 and D225. Data for PIZV control were collected for a previous Indian rhesus macaque study described by Young et al [1]. Serum samples were collected every month (D0, D29, D57, D85, D113, D14, D169, D225, D253, D281, D365, D401, D476, D490) to determine mean neutralizing antibody titers (EC_50_). Data analysis was performed in Graph Pad Prism 7 as described under statistical analysis section. Treatment groups were compared by 1-way ANOVA and Tukey’s Multiple Comparison in GraphPad Prism. All animals that received PIZV elicited a neutralizing antibody response against PIZV. No animals were excluded from data analysis. Serum samples from all groups were used to determine the specificity of the Z-RVP-384 assay.

Macaques were monitored at least twice daily for stress or suffering, and body weight was measured on D1 prior to dosing and monthly thereafter. Cageside observations included observation for mortality, moribundity, general health, and signs of toxicity. Treatment with the vaccines had no effect on mortality, physical examinations, cageside observations, body weights or body weight changes. Moribund animals and all surviving animals at the end of the study were euthanized by intravenous injection of sodium pentobarbital (or equivalent) and exsanguinated.

**Flavivirus Naïve rhesus macaque study**

Rhesus macaque sera were obtained from a previously published PIZV vaccination and ZIKV challenge study conducted at either Charles River Laboratories (CRL), Mattawan. MI. All in-life practices, including husbandry and environmental enrichment, were approved and conducted per IACUC of CRL under protocols #2715-001 and #2715-002. Details of the study are described in the published paper [1].

**Cynomolgus macaque study**

Cynomolgus macaque sera were obtained from a previously published ZIKV challenge study conducted at Wisconsin National Primate Research Center. Madison, WI USA. All in-life practices, including husbandry and environmental enrichment, were approved and conducted per IACUC of The University of Wisconsin-Madison, College of Letters and Science and Vice Chancellor for Research and Graduate Education Centers Institutional Animal Care and Use Committee under protocol number G005401-R01. The details of the study are described in the published paper [2].

**Reference**

1. Young G, Bohning KJ, Zahralban-Steele M, Hather G, Tadepalli S, Mickey K, et al. Complete Protection in Macaques Conferred by Purified Inactivated Zika Vaccine: Defining a Correlate of Protection. Sci Rep. 2020;10(1):3488.

2. Breitbach ME, Newman CM, Dudley DM, Stewart LM, Aliota MT, Koenig MR, et al. Primary infection with dengue or Zika virus does not affect the severity of heterologous secondary infection in macaques. PLoS Pathog. 2019;15(8):e1007766.
